# Electronic cigarettes and cardiovascular disease: epidemiological and biological links

**DOI:** 10.1007/s00424-024-02925-0

**Published:** 2024-02-20

**Authors:** Huiqi Zong, Zhekai Hu, Weina Li, Mina Wang, Qi Zhou, Xiang Li, Hongxu Liu

**Affiliations:** 1 Beijing Hospital of Traditional Chinese Medicine, Capital Medical University, Beijing, 100010 China; 2 Beijing University of Chinese Medicine, Beijing, 100029 China; 3 Guang’anmen Hospital, China Academy of Chinese Medical Sciences, Xicheng District, Beijing, 100053 China

**Keywords:** e-cigarettes, Cardiovascular disease, Smoking, Vaping, Toxicity

## Abstract

Electronic cigarettes (e-cigarettes), as alternative nicotine delivery methods, has rapidly increased among youth and adults in recent years. However, cardiovascular safety is an important consideration regarding e-cigarettes usage. e-cigarette emissions, including nicotine, propylene glycol, flavorings, nitrosamine, and metals, might have adverse effects on cardiovascular health. A large body of epidemiological evidence has indicated that e-cigarettes are considered an independent risk factor for increased rates of cardiovascular disease occurrence and death. The incidence and mortality of various types of cardiovascular disease, such as cardiac arrhythmia, hypertension, acute coronary syndromes, and heart failure, have a modest growth in vapers (users of e-cigarettes). Although the underlying biological mechanisms have not been fully understood, studies have validated that oxidative stress, inflammation, endothelial dysfunction, atherosclerosis, hemodynamic effects, and platelet function play important roles in which e-cigarettes work in the human body. This minireview consolidates and discusses the epidemiological and biological links between e-cigarettes and various types of cardiovascular disease.

## Introduction

Smoking is one of the most common health risks among various chronic diseases [1]. In the United States, smoking is the number one cause of cardiovascular disease (CVD) [2], resulting in 440,000 deaths every year [3]. There are nearly 13 billion smokers worldwide, of which nearly 80% live in developing countries [4]. At present, epidemiological studies emphasize that smoking leads to the initiation and development of CVD [5]. Many leading tobacco control researchers and policymakers believe that the most important goal in reducing the harmful effects of tobacco use should be to reduce or eliminate the use of combusted tobacco [6, 7]; thus, electronic cigarettes (e-cigarettes) emerged.

e-cigarettes have been launched since 2006 with increased sales, particularly in Europe and the USA [8] due to the less-toxic promotion and unique tastes. A survey on e-cigarettes in the UK showed that the use of disposable e-cigarettes in British grew rapidly between 2021 and 2022, especially among younger adults, and further demonstrated most young adult vapers (users of e-cigarettes) in British now use disposable products [9]. In 2019, it was reported that about one-third of adult smokers in the United States used e-cigarettes (over 10 million) [10]. In addition, it attracted a large group of adolescent vapers: 22% of high school students and 9.4% of middle school students report daily use of e-cigarettes [11]. With the increasing use of e-cigarettes, the American Lung Association, World Health Organization, American Association of Pediatrics, the US Centers for Disease Control, and Prevention are gradually paying attention to e-cigarettes. These data indicate that e-cigarettes not only attract smokers who are attempting to quit but are popular among nonsmokers, with users gradually becoming younger.

As noncombusted products, e-cigarettes can reduce exposure to combustion-generated toxic substances. Due to the simplification and improvement of aerosol formation modes, the properties and concentration of e-cigarette aerosol differ from tobacco smoke, further indicating that the toxicity of e-cigarette smoke is much lower than that of tobacco cigarette smoke [12]. Despite a lot of evidence showing e-cigarettes have benefits and can help quit smoking, others argue against e-cigarettes raise various concerns, including its health hazards; Inducing teenagers to smoke e-cigarettes, making the smoking population younger; dual use with tobacco cigarettes may reduce rates of cigarettes smoking cessation [13, 14]. Heightened concern over the usage safety of e-cigarettes from these studies displays further investigations need to be carried out to better understand the CVD effects of using e-cigarettes. In this review, we provide an overview of the potential adverse effects of e-cigarettes on cardiovascular health by describing the operation, components, and potential mechanisms of cardiovascular toxicity of e-cigarettes. Furthermore, based on existing human and animal research data, we evaluated the potential cardiovascular hazards of e-cigarettes in order to provide guidance for clinical practice.

## Design of e-cigarette device (Fig. 1)

There are various forms of e-cigarettes since it has been first introduced [15]. Common forms are first-generation (cigarette-like), second-generation (rechargeable), and third-generation (personalized tank) devices. The first generation of devices not only resembled tobacco cigarettes in appearance but also had disposable cartridges. The second generation is improved than the first generation, and the cartridge is larger. It contains both the e-liquid and the heating coil, which will produce a large amount of aerosols and release higher doses of nicotine. The third-generation equipment has replaceable heating coils, wicking materials, e-liquids, and batteries [16]. e-cigarette users can adjust the nicotine concentration by changing the temperature, voltage, or power applied to the atomizer through third-generation devices, which provides great convenience. Advanced generation e-cigarettes typically hold more amounts of e-liquid, produce more aerosols, and release more nicotine than previous generation e-cigarettes, allowing users to inhale more concentrated puff volumes in a short period of time, which indicates additional generation may pose a greater threat to health. Despite the diversity of their designs, all e-cigarettes have similar function, typically consisting of three major parts: a cartridge or reservoir filled with an e-liquid solution, a heating element and a lithium battery power source [17], which are activated by inhalation or pressing a button. With inhalation, the airflow triggers the atomizer, and the e-liquid is vaporized [18], producing a vapor mist that carries nicotine vapor [19].

**Fig. 1:**
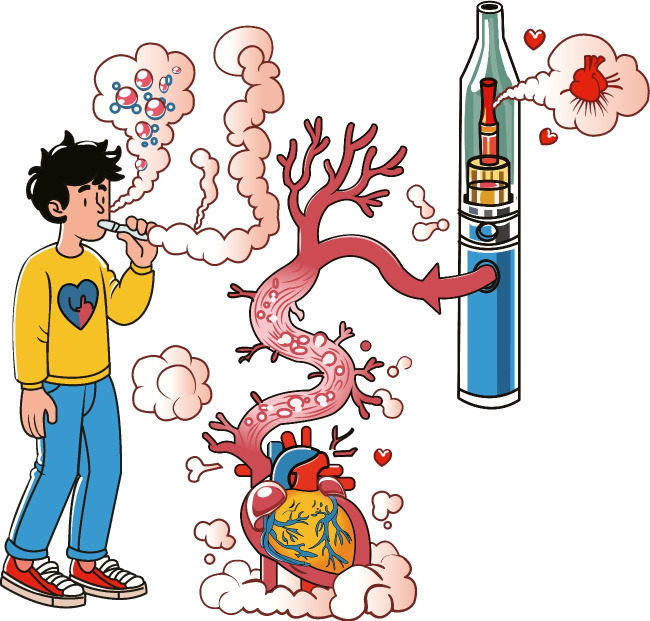
A cartoon describing the vaporization process of e-cigarette. Here is a cartoon-style comic illustrating the vaporization process of e-cigarette and its relation to cardiovascular diseases(CVDs). This comic provides a visual narrative, starting from the use of an e-cigarette to the potential harmful effects on the cardiovascular system. In the upper left part of the cartoon, we can see that e-cigarette vapor(ECV) contains many substances such as nicotine, flavorings, aldehydes, and metals, which may be harmful to human. In the upper right part of the cartoon is the design of an e-cigarette device. On the right side of the cartoon is the adverse effects of ECV on blood vessels and heart. Through mechanisms such as oxidative stress, inflammation, and endothelial dysfunction, ECV can increase heart rate, blood pressure, and clog blood vessels

## The toxic components of e-cigarettes

**Fig. 2:**
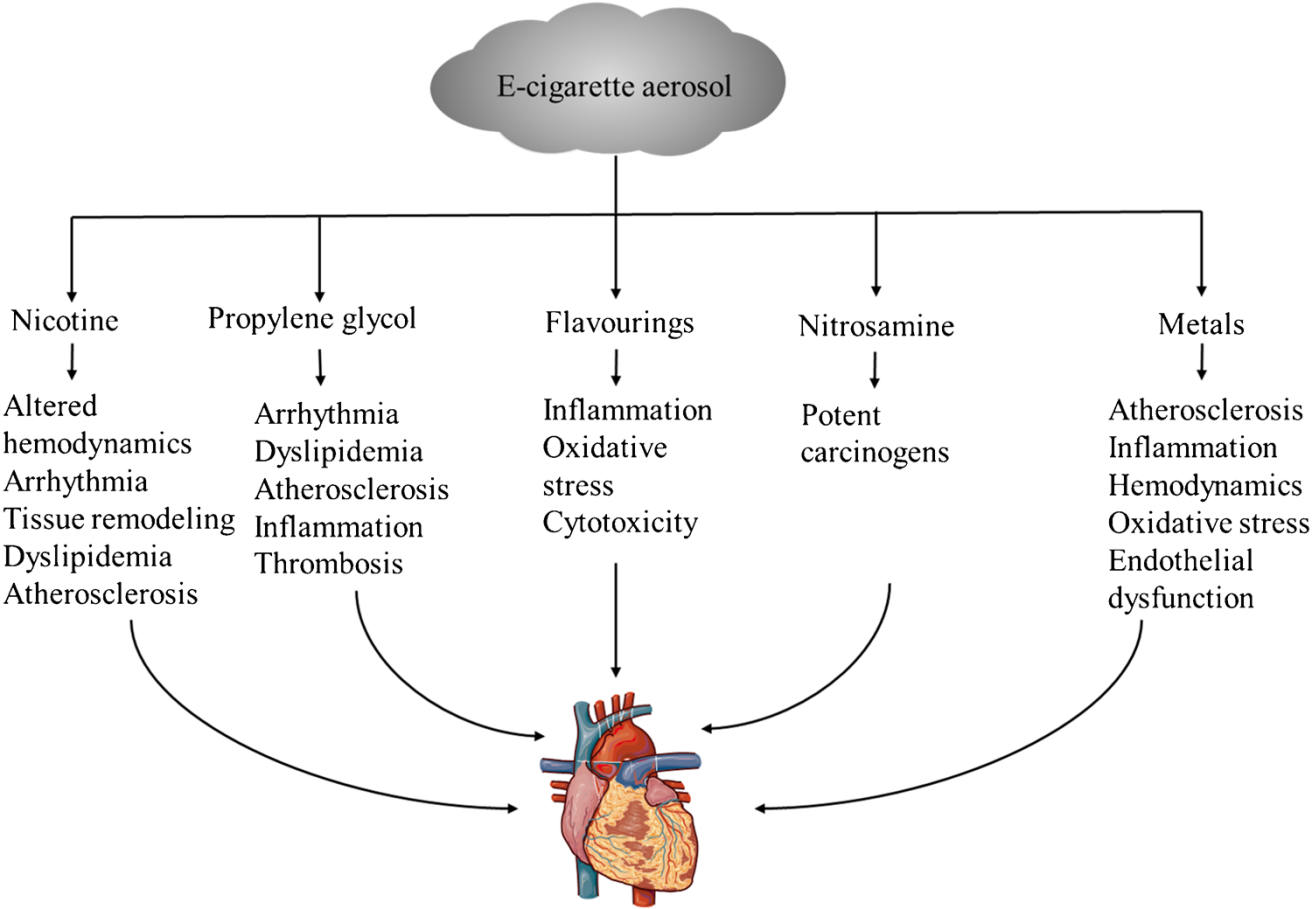
Potential adverse cardiovascular effects induced by various constituents of e-cigarette aerosol

Due to the simplification of the tobacco combustion process, the composition of e-cigarettes liquid is also much simpler than that of tobacco cigarettes. e-cigarette liquid mainly includes 3 substances: propylene glycol, vegetable glycerin as the carrier solvents, and nicotine. At the same time, e-cigarettes contain other substances, such as flavor additives, N-nitrosamines, and volatile organic compounds [20] detected in e-cigarettes, as well as metal contamination through heating coils, solders, and wicks, such as cadmium, chromium, lead, nickel, silver, tin, and silicates [20]. These compounds present an added layer of complexity when evaluating the cardiovascular toxicity of e-cigarettes [21]. Furthermore, the concentration of these compounds varies with the manufacturer of the e-cigarettes liquid, the type of e-cigarettes, the coils temperature, and the airflow rate [22]. The potential cardiovascular effects related to these substances are summarized in Fig. 2.

### Nicotine

Compared to conventional cigarettes, e-cigarettes contain less chemicals, but most do contain nicotine which is the most important risk factor and has a number of adverse effects on the cardiovascular system. The effects of nicotine on systemic hemodynamics are mainly mediated by activation of the sympathetic nervous system [23]. Nicotine releases norepinephrine from adrenergic neurons and increases adrenal release of epinephrine [6]. e-cigarettes can increase plasma epinephrine, thereby stimulating heart rate, increasing blood pressure, and ultimately promoting the increase of cardiac output [6]. A recent study found significant relevance between smoking and hypertensive heart disease and hypertensive nephropathy [24]. Because beta adrenaline can stimulate tissue remodeling, nicotine promotes myocardial remodeling through sustained sympathetic activation, thereby increasing the risk of heart failure [23]. Additionally, catecholamine released from nicotine could lead to fatal ventricular tachycardia and atrial fibrillation. Perhaps this is why smokers have an increased risk of sudden cardiac death. On average, compared with nonsmokers, smokers have lower levels of high-density lipoprotein cholesterol and higher levels of low-density lipoprotein cholesterol [6]. Nicotine induces lipolysis through catecholamine action at β-adrenoreceptors and increases plasma-free fatty acid concentrations, which may contribute to enhanced synthesis of low-density and reduction of high-density lipoprotein [6, 25]. Therefore, dyslipidemia exists among most smokers. In general, smoking is considered a major risk factor for atherosclerosis, and nicotine accelerates the progress of atherosclerosis [26]. The induction of this process may be associated with the growth factors produced by vascular endothelial cells, such as platelet-derived growth factor and vascular endothelial growth factor [27]. However, the effect of nicotine on platelet activity has always been controversial. Some studies suggested that nicotine may regulate platelet activation by decreasing aggregation, thereby decreasing thrombosis [28], while other studies showed that nicotine has no effect on platelet activity [29]. More experiments are needed to prove the relationship between nicotine and thrombogenesis. Smoking can cause intermittent peaks and troughs of nicotine in the blood. Compared to smokers, e-cigarette users tend to smoke more evenly throughout the day, thereby reducing nicotine levels in the blood, leading to less harm to the human body [30]. As for the content of nicotine in e-cigarettes, it is determined by the type and concentration of e-cigarettes. The cardiovascular toxicity of nicotine also depends on different e-cigarette devices.

### Propylene glycol and vegetable glycerin

As the most common constituents in e-liquid formulations, propylene glycol and glycerol can produce toxic emissions when heated. The heating procedure of propylene glycol can originate plenty of thermal dehydration products, mainly including acetaldehyde, formaldehyde, propylene oxide, acetol, allyl alcohol, glyoxal, and methylglyoxal [31]. While glycerol can generate acrolein and formaldehyde, as well as dehydrated glycerol. Cardiomyocytes are very sensitive to the toxic effects of formaldehyde. Research has found [32] that formaldehyde is an important toxicant that causes cardiovascular toxicity, causing many CVDs such as atrioventricular block, arrhythmia (including sick sinus syndrome), ventricular tachycardia, ventricular fibrillation, and atrial fibrillation. As a reactive a,b-unsaturated aldehyde, acrolein can exacerbate dyslipidemia that can lead to accelerated atherosclerosis, modify A-I protein in high-density lipoprotein (HDL) [33, 34] and catalytic neutrophil-mediated inflammation [35], and enhance platelet activation which contributes to an increased risk of thrombosis in animal models [36]. Most notably, formaldehyde and acrolein exhibit their toxicity through adduct formation with proteins and deoxyribonucleic acid (DNA) [37]. Marin et al. [37, 38] found that an acrolein concentration in the lung of an e-cigarette user is 16-fold higher than that in the breathing air recommended World Health Organization (WHO) threshold and 320-fold higher than the US Environmental Protection Agency (EPA) threshold. This indicates that the concentration of acrolein produced by e-cigarettes significantly exceeds the normal level, posing a threat to human health. Even so, in a cross-sectional study, Shahab et al. found that the levels of acrolein in e-cigarette-only users were significantly lower than those in combustible cigarette-only users [39]. In addition, acetaldehyde has been proven to cause alcoholic cardiomyopathy through mitochondrial dysfunction in mice [40]. The cardiovascular toxicity of these aldehydes can vary significantly depending on the heating procedure. Increasing the battery voltage from 3.3V to 4.8V doubles the vaporization amount of e-liquid, accompanied by a significant increase in the generation and emissions of total aldehyde [31]. Under normal circumstances, e-cigarettes seem to be safer than tobacco cigarettes. However, at high battery voltages, aldehyde emissions are closer to and could even exceed those generated by cigarettes [41]. Reuse of a device can also increase aldehyde generation [41], which is believed to be related to the accumulation of polymerization products that degrade when heated.

### Flavorings

Flavorings, with their unique taste, are the main reason that attracts youth (age 12–17 years) to use e-cigarettes [42]. “comes in flavors that I like” was the reason for the highest ranking among youth who were e-cigarettes vapers [43]. All brands of e-cigarettes were reported to have over 7700 unique flavors [44], and these flavoring chemicals added a layer of complexity to the toxicity of e-cigarettes. Flavorings may contain mixtures of alcohol, terpenes, and aldehydes, as well as toxic chemicals such as diacetyl and benzaldehyde, which can lead to pulmonary injury [45]. Additionally, monoamine oxidase inhibitors present in e-liquids, such as vanillin and ethylvanillin, have been proven to enhance nicotine addiction in smokers by delaying the catalytic degradation of monoamine oxidase enzymes to neurotransmitters [46]. Muthumalage et al. found the flavorings in e-liquids can trigger an inflammatory response in monocytes, mediated by reactive oxygen species (ROS) production, which leads to dose-dependent cytotoxicity [47]. While after treating with various flavoring compounds, the endothelial cells of the subjects decreased the production of nitric oxide and induced inflammation consistent with endothelial dysfunction [48]. It is reported that at least 65 individual flavoring ingredients in flavored e-liquids were noxious, while cinnamaldehyde was most commonly observed to be cytotoxic, followed by vanillin, menthol, ethyl maltol, ethyl vanillin, benzaldehyde, and linalool [49]. In general, these flavoring studies highlight the need for regulations that prohibit toxic flavors and restrict their concentration in e-cigarettes.

### Nitrosamine

After using e-cigarettes, nicotine is rapidly metabolized into cotinine and nitrosamines. Cotinine is metabolized with nontoxicity by entering the urine eventually [50]. Inhaled nitrosamines are believed to be potent carcinogens that can induce tumors in different organs of animal models [51]. Therefore, the content and level of nitrosamines in blood fluid have been regarded as a gold standard for determining the potential carcinogenicity of smoking [52]. Shahab et al. [39] found vapers have lower levels of nitrosamines than smokers, but are significantly higher than nonsmokers, which indicates that e-cigarettes are potentially carcinogenic.

### Metals

e-cigarettes can generate aerosols by heating e-liquids with metal coils [53]. The presence of metals and metalloids (e.g., arsenic, chromium, lead, and nickel) in e-cigarette aerosols causes many adverse effects in different organs of humans, such as CVD [54], pulmonary injury and renal damage [55]. Generally, metals/metalloids exist in soldered joints [56] and coils, which are made of alloys or high-purity metals. Heating coils are an important way to promote toxic metals exposure. If coils exceed 1000°C [57], coils degrade rapidly and facilitate the release of metals. If 150 heat cycles were performed, chromium and iron from Kanthal coils would be lost up to 19% and 58%, respectively, and iron and nickel from nichrome coils up to 14% and 43%, respectively [58]. Studies [55] have found most metal/metalloid biosample levels in e-cigarette users were similar or even higher in comparison with tobacco cigarette users and higher in comparison with cigar users. Therefore, regulation is necessary to show the potential for contact with metal/metalloid exposure during use and provide detailed information on the hazards of heating these metals for e-cigarette users.

## The association between e-cigarettes and various CVDs (Fig. 1)

### Association between e-cigarettes and cardiac arrhythmia

Cardiac arrhythmias, due to the high cost of diagnostic and therapeutic interventions, are a major public health problem in low and middle-income countries [59]. The clinical manifestations of patients with heart rhythm abnormalities vary from a lack of symptoms to sudden cardiac arrest, and even leading to sudden cardiac death (SCD). SCD, which is a primary cause of death worldwide, accounts for 50–60% of deaths in patients with coronary artery disease [60, 61]. Recent studies have suggested that e-cigarettes are strongly associated with cardiac arrhythmias, pointing out an independent risk factor for cardiac arrhythmias. A cross-sectional study showed that e-cigarette users have a higher risk of coronary heart disease, arrhythmia, chest pain, or palpitations [62]. Carll et al. [63] found that e-cigarette aerosols, from e-cigarette solvents (vegetable glycerin and propylene glycol), induced arrhythmia, impaired ventricular repolarization, and stimulated autonomic reflexes, ultimately leading to cardiac risk. Due to the higher levels of acrolein and formaldehyde [64] in the aerosols from menthol-containing e-cigarettes, this flavor of e-cigarettes is more likely to increase the likelihood of ventricular premature beats than tobacco-flavored e-cigarettes. Notably, e-cigarette solvent-induced bradyarrhythmias and bradycardia were more pronounced in male than in female mice, which indicates that males may be more sensitive to the direct cardiac impacts of e-cigarette solvents. Importantly, chronic repetition of e-cigarettes can lead to increased arrhythmia and prolonged QT [65]. Those who suffer from Long QT Syndrome are more likely to succumb to sudden cardiac death predominately in their youth [65]. In general, these results show that e-cigarette use could trigger arrhythmias by inducing autonomic imbalance, and the magnitude and characteristics of the reaction may depend on chemicals in the e-liquids, such as nicotine, solvents, and flavors.

### Association between e-cigarettes and hypertension

Hypertension is a leading modifiable cause of CVD mortality and disease burden in most parts of the world, which is also one of the risk factors for stroke or other CVDs [66, 67]. As reported [68], the number of hypertensive patients worldwide exceeded 1 billion in 2019, with a prevalence rate of 32% for females and 34% for males aged 30–79, which indicates that hypertension is very common among the population. The association between e-cigarettes and hypertension has been widely reported. Interestingly, Hua et al. [69] collected short-term health effects produced by e-cigarette use from three online e-cigarettes forums and found that negative impacts account for the majority (82%), with hypertension being the most common cardiovascular symptom diagnosed by physicians. Hypertension caused by e-cigarettes may be due to the release of nicotine. Nicotine releases norepinephrine and epinephrine, activating the sympathetic nervous system. High-adrenaline concentrations induce vasoconstriction and increased cardiac contractility through activation of α1 adrenoreceptors and β1 adrenergic receptors, respectively [70], eventually leading to elevated blood pressure. When using e-cigarettes, the tiny nicotine particles contained in the vapor in a fast and effective way into the bloodstream, resulting in a plasma nicotine concentration, which may ultimately increase the risk of hypertension [71]. In addition, inhaled aerosols of acetaldehydes and propionaldehydes activate the sympathetic nervous system by stimulating the release of catecholamines, thereby elevating blood pressure in mice models [72]. However, a study showed regular e-cigarette use may aid smokers with arterial hypertension control blood pressure [73]. This is contrary to the previous results, highlighting the uncertainty of long-term health consequences of vaping. We need more research to demonstrate the impact of e-cigarettes on blood pressure, but dual smokers of e- and tobacco cigarettes certainly have a higher prevalence of hypertension [74]. As a result, e-cigarettes are a modifiable risk factor and its association with hypertension cannot be ignored.

### Association between e-cigarettes and acute coronary syndromes

Despite the fact that significant progress has been made in the diagnosis and treatment of acute coronary syndromes (ACS), CVD is considered to be the leading cause of death worldwide, with approximately half of these deaths as a result of ischemic heart disease (IHD) [75, 76]. As an important component of IHD mortality, myocardial infarction (MI) show an increased trend in the hospitalization incidence, morbidity, and mortality rate [77, 78]. The correlation between e-cigarettes and an elevated risk of ACS has been verified in many studies. Interestingly, in a randomized trial on smoking cessation treatments after ACS hospitalization (*n*=49), Busch et al. [79] found if participants were to regularly use only e-cigarettes, their perceived risk of developing a heart attack next year was 34.6%, significantly lower than the perceived risk of regularly smoking only tobacco cigarettes (56.2%) and significantly higher than the perceived risk of no-nicotine use (15.2%). In a National Health Interview Survey (NHIS) in America, Alzahrani et al. [80, 81], using years 2014 (*n*=36,697), 2015 (updated in a subsequent letter to the editor), and 2016 (*n*=33,028) data, reported an odds ratio (OR) of 1.49 (95% confidence interval (CI), 1.06–2.09) for occasional e-cigarette users and 2.14 (95% CI, 1.41–3.25) in those vaping daily, which indicates high cumulative risk estimates for MI. They concluded that daily e-cigarette use was independently associated with increased odds of having had a MI. Similarly, Vindhyal et al. [82], analyzing the NHIS data of 2014 (*n*=36,697), 2016 (*n*=33,028), and 2017 (*n*=26,742), found e-cigarette users have an over 50% higher risk of developing MI (OR, 1.56; 95% CI, 1.45–1.68) and even a 30% higher risk of developing stroke (OR, 1.30; 95% CI, 1.20–1.40) when compared with nonusers. In addition, Osei et al. [83] found dual users were associated with 36% higher odds of CVD (OR, 1.36; 95% CI, 1.18–1.56) compared with smoking alone in a large cross-sectional telephone survey. A composite of self-reported coronary heart disease, MI, or stroke was regarded as the main outcome. They also found the odds of CVD gradually increased with increasing frequency of e-cigarette exposure in current tobacco cigarette smokers. The exposure to e-cigarette vapor extracts enhances platelet activation, adhesion, and aggregation [84–86], which is associated with MI [85–87]. Taken together, we conclude that significant associations exist between e-cigarettes and ACS, which indicates e-cigarettes serve as an independent risk factor for ACS. The impact of e-cigarettes on ACS also depends on the dosage and may magnify using “traditional” combustible cigarettes simultaneously.

If patients do not quit smoking promptly, e-cigarettes can also worsen heart failure [88]. In short, the links between e-cigarettes and CVDs are close. Table 1 provides a nonexhaustive list of association studies between CVDs and e-cigarettes.

**Table 1:** The association between e-cigarettes and various CVDs

CVDs	References	Study object	Findings
Cardiac arrhythmia	Wang et al. [62] 2018	E-cigarette users in the Health eHeart Study	E-cigarette users have a higher risk of arrhythmia
	Carll et al. [63] 2022	C57BL6/J mice	e-cigarettes and their lone constituents induce cardiac arrhythmia in mice
	Conklin et al. [64] 2018	C57BL6/J mice	Menthol-containing e-cigarettes are more likely to increase the risk of ventricular premature beats
Hypertension	Hua et al. [69] 2013	E-cigarette users in online forums	Hypertension is the most common cardiovascular symptom after using e-cigarettes
	Egle et al. [72] 1972	Male Wistar rats	Inhaled acetaldehyde and propionaldehyde in e-cigarettes can elevate blood pressure in mice models
	Polosa et al. [73] 2016	Hypertensive smokers who quit or reduced tobacco consumption by switching to e-cigarettes	e-cigarette use may aid smokers with arterial hypertension control blood pressure
	Kim et al. [77] 2022	275,762 participants from the Korea Community Health Survey	Dual smokers of e-cigarettes and tobacco cigarettes certainly have a higher prevalence of hypertension
Acute coronary syndromes	Busch et al. [79] 2016	Participants were drawn from a randomized trial of smoking cessation treatments following hospitalization for ACS	The risk of using e-cigarettes regularly was lower than regularly smoking only, while higher than no-nicotine use
	Alzahrani et al. [80, 81] 2018,2019	Using years 2014, 2015, and 2016 data from The National Health Interview Surveys	Daily e-cigarette use increased odds of MI
	Vindhyal et al. [82] 2019	Analyzing the NHIS data of 2014, 2016, and 2017	e-cigarette users have an over 50% higher risk of developing MI
	Osei et al. [83] 2019	Using 2016 and 2017 data of 449,092 participants from the Behavioral Risk Factor Surveillance System	The odds of CVD gradually increased with increasing frequency of e-cigarette exposure in current combustible-cigarette smokers
	Kivrak et al. [87] 2014	Case-report A healthy 24-year-old man	A man developed acute myocardial infarction after only 1 month of e-cigarette use
Heart failure	Gathright et al. [88] 2020	Data from the Population Assessment of Tobacco and Health Study	e-cigarettes can worsen heart failure

*ACS* acute coronary syndromes, *CVD* cardiovascular disease, *MI* myocardial infarction, *NHIS* National Health Interview Surveys

## Biological links between e-cigarettes and CVDs (Table 2)

### Oxidative stress

Oxidative stress, regarded as the initiating factor of aging and a variety of diseases, is an imbalance state between the production of reactive oxygen species (ROS) and the endogenous antioxidant defense mechanisms [99]. In the process of development, oxidative stress may activate the antioxidant defense system, release inflammatory cytokines, and produce oxidative mediators. Oxidative stress has also been proven to play an important role in CVDs. On the one hand, oxidative stress can result in cardiac fibrosis by stimulating the proliferation of cardiac fibroblasts which is responsible for extracellular matrix remodeling [100]. On the other hand, oxidative stress interferes with nitric oxide synthase by inhibiting the Nuclear Factor erythroid 2-Related Factor 2 (Nrf2) pathway and upregulating asymmetric dimethylarginine content. The dysregulation of nitric oxide synthase and decoupling of endothelial nitric oxide synthase (eNOS) eventually lead to endothelial dysfunction, which is considered the pathological basis of atherosclerosis, hypertension, and cardiomyopathy [101]. In addition, oxidative stress also activates vascular smooth cells by promoting their proliferation and migration, which plays a crucial role in vascular calcification and remodeling [102]. A cross-sectional case-control study found that habitual e-cigarette use was associated with the transition from cardiac autonomic balance to sympathetic predominance. Compared with nonuser controls, low-density lipoprotein (LDL) oxidizability and oxidative stress-related biomarkers were increased in e-cigarette users [89], which indicates chronic e-cigarette use is associated with increased cardiovascular risk. In a comprehensive study, Kuntic et al. [90] assessed the effects of e-cigarette vapor (ECV) on vascular function in smokers and experimental mice. They found ECV exposure-induced inflammation and vascular, cerebral, and pulmonary oxidative stress, as well as increased blood pressure in mice. These studies elucidate that exposure to ECV may lead to oxidative stress, which potentially induces marked adverse effects on CVDs.

**Table 2:** Biological links between e-cigarettes and CVDs

Key events	References	Study object	Effects
Oxidative stress	Moheimani et al. [89] 2017	Habitual e-cigarette users and nonuser control individuals from 2015 to 2016 at the University of California, LA	Oxidative stress; a shift in cardiac autonomic balance toward sympathetic predominance
	Kuntic et al. [90] 2020	20 healthy subjects; C57BL/6 mice	Oxidative stress, inflammation, increased blood pressure
Inflammation	Higham et al. [91] 2016	Blood samples from ten healthy nonsmokers	Inflammation in human neutrophils
	Stokes et al. [92] 2021	Blood and urine samples from adults aged 18+ years in the PATH study	Tender inflammatory response; oxidative stress
Endothelial dysfunction	Antoniewicz et al. [93] 2016	Blood samples from sixteen healthy seldom smokers	Increased endothelial progenitor cells
	Anderson et al. [94] 2016	Human umbilical vein endothelial cells	Oxygen species, DNA damage, the reduction of cell viability
	Putzhammer et al. [95] 2016	Human umbilical vein endothelial cells	High cytotoxicity, inhibition of cell proliferation, altered cell morphology
Atherosclerosis	Li et al. [96] 2021	ApoE^−/−^ mice models	Progression in atherosclerosis
	Derout et al. [97] 2019	ApoE^−/−^ mice models	Atherosclerosis, oxidative stress, mitochondrial DNA mutations
Platelet function	Qasim et al. [86] 2018	C57BL/6 mice	Increase the risk of thrombogenic events; enhance platelet function
	Hom et al. [98] 2016	Fresh PRP pheresis packs were obtained from the Oklahoma Blood Institute, from healthy nonsmoking donors	The activation, aggregation, and adhesion of platelets were enhanced

*ApoE*^*−/−*^ *mice* apolipoprotein-E knockout mice, *PATH* Population Assessment of Tobacco and Health Study, *PRP* platelet-rich plasma

### Inflammation

Inflammation is the defensive response of an organism to external stimuli; however, inflammation is also considered a pathological basis for various diseases by injuring tissues. For example, chronic inflammation could accelerate atherosclerosis and induce plaque instability, causing acute cardiovascular events. When inflammation occurs, multiple inflammatory signaling pathways are activated, such as p38 MAP kinase (p38 MAPK), phosphatidylinositol-3-kinase (PI3K), and janus kinase/signal transducer and activator of transcription (JAK/STAT) [103]. Higham et al. observed that ECV extract caused an increase in neutrophil elastase activity and p38 MAPK activation, which indicates e-cigarette exposure leading to a pro-inflammatory response in human neutrophils [91]. In a longitudinal cohort study, Stokes et al. [92] used nationally representative data to identify the relationship between cigarettes or e-cigarette exposure and biomarkers of inflammation and oxidative stress. They found no difference in inflammatory and oxidative stress biomarkers between only e-cigarette users and nonusers (no smoking or vaping), and levels were lower in only e-cigarette users compared to only smokers. It proved that e-cigarette exposure induced a tender inflammatory response than tobacco cigarettes, which supports that e-cigarettes can become an alternative tool for smoking cessation. Of course, the concentrations and contents in e-liquids and vapors may have different effects once heated, atomized, and metabolized in vivo settings, so we should be cautious to interpret these studies. We also need more research to determine the inflammatory mechanisms of e-cigarette exposure.

### Endothelial dysfunction

As the continuous inner cell wall of the cardiovascular system, vascular endothelium plays an important role in maintaining this homeostatic network [104]. Endothelial cell dysfunction broadly refers to a variety of nonadaptive alterations in functional phenotype, which is responsible for adverse effects on vascular responsiveness, such as hemostasis, thrombosis, and inflammatory reactions in the arterial wall. At present, endothelial cell dysfunction is the earliest detectable change in the development of an atherosclerotic lesion, which may contribute to a series of CVDs including vascular inflammation, hypertension and cardiomyopathy [105]. In addition, elevated endothelial progenitor cell (EPC) levels can serve as a biomarker of vascular injury [18]. Antoniewicz et al. [93] determined vascular changes by measuring EPCs and microvesicles among healthy young volunteers after a short period of exposure to ECV. They found EPC levels in blood were significantly increased 1 hour after exposure to ECV and returned to baseline values 24 hours later, which suggests vascular may injure following short e-cigarette inhalation. Anderson et al. experimented on the effects of human umbilical vein endothelial cells (HUVECs) to e-cigarette exposure and found that e-cigarette aerosol could induce reactive oxygen species (ROS), cause DNA damage, and significantly reduce cell viability in a concentration-dependent manner. This further indicated that electronic aerosols may contribute to cell apoptosis and programmed necrosis [94]. Putzhammer et al. [95] evaluated toxicity on ECV from different e-cigarette brands. They found some ECV extracts inhibited cell proliferation and altered cell morphology, eventually leading to endothelial cell dysfunction. Of course, compared to tobacco cigarette smoke extracts, ECV has a slightly lighter impact on endothelial cells. From these studies, we have concluded that e-cigarettes can indeed result in endothelial dysfunction, although the severity is not as severe as tobacco cigarettes. Vascular endothelium cell dysfunction, which is regarded as the pathological basis of CVDs, is a central or exacerbating component in smoking/vaping-related pathologies. Therefore, this mechanism helps us better understand the cardiovascular toxicity of e-cigarettes.

### Atherosclerosis

At the early stage of atherosclerosis (AS), it selectively recruits circulating monocytes from the blood into the intima, then activates inflammation. At this moment, monocytes differentiate into macrophages that internalize modified lipoproteins and turn into foam cells, which is the hallmark of forming early fatty streak lesions. Subsequently, various chemokines and growth factors refined by activated endothelial cells and macrophages act on adjacent smooth muscle cells (or their precursor 12), inducing their proliferation and synthesis of extracellular matrix components in the intimal compartment, resulting in fibromuscular plaques [104]. The slow formation of atherosclerotic plaque is mostly asymptomatic, but plaque rupture or endothelial erosion will trigger thrombosis, leading to an atherothrombotic occlusion [104, 106]. This complex process ultimately contributes to CVDs such as myocardial infarction or ischemic stroke [107]. Numerous studies have reported the pro-atherosclerotic effects induced by e-cigarette exposure. Li et al. [96] revealed the association between e-cigarettes and atherosclerosis in apolipoprotein-E knockout (ApoE^−/−^) mice models. They concluded ECV can elevate level of damaged mitochondrial DNA in circulating blood and induce the expression of toll-like receptor 9 (TLR9), thereby increasing the expression of proinflammatory cytokines in monocyte/macrophage, which is responsible for atherosclerosis. Furthermore, Derout et al. [97] demonstrated increased oxidative stress, mitochondrial DNA mutations and atherosclerosis in the aortic root of ApoE^−/−^ mice following exposure to e-cigarettes with 2.4% nicotine. Taken together, e-cigarette exposure promotes the progression of atherosclerosis. However, most of these results come from animal experiments, suggesting the need for more validation from human studies.

### Hemodynamic effects

The systemic hemodynamic effects of e-cigarettes, which primarily stem from nicotine, are mediated by activation of the sympathetic nervous system. Sympathetic stimulation is the result of activation of actions of nicotinic cholinergic receptors in the peripheral nervous system and central nervous system. Nicotine contributes to an increase in plasma epinephrine release, manifested by increased heart rate, enhanced myocardial contractility and elevated blood pressure [23]. Therefore, the systemic hemodynamic effect of e-cigarettes is equivalent to the effect of nicotine on hemodynamics [18]. However, differences in device-specific delivery of nicotine may result in variability in effects across studies. Some new devices with lower resistance, higher voltage, and e-liquid nicotine concentration produce nicotine levels in the blood comparable to tobacco cigarettes [108, 109].

### Platelet function

Platelets are the primary cell maintaining vascular homeostasis. In the process of the initial hemostasis, platelets adhere to the damaged blood vessel wall at the site of injury, which is completed through multiple signaling cascades [110]. In addition, platelets actively participate in immune responses to microorganisms and foreign substances. Through specific receptors and granule release, RNA transfer, and mitochondrial secretion, platelets play an irreplaceable role in regulating hemostasis and thrombosis, infection, and innate and adaptive immunity [111]. Therefore, abnormal platelet function can lead to venous thromboembolism, myocardial infarction and stroke [111]. Vaping is one of the most modifiable risk factors for thrombosis. Qasim et al. [86] evaluated the effects of e-cigarettes and clean air on platelet function and thrombogenesis in mice models via a vapor inhalation system. Their results showed that platelets from e-cigarette-exposed mice were overactive, exhibiting enhanced aggregation, resisting to inhibition by prostacyclin compared with clean air-exposed platelets. It was demonstrated that exposure to ECV extracts can produce a shortened thrombosis occlusion and bleeding times. Furthermore, Hom et al. [98] evaluated the effects of tobacco smoke extracts, ECV extracts, and pure nicotine on platelet activities. Interestingly, they found that platelets from exposure to ECV extracts caused remarkable up-regulation in proinflammatory markers of gC1qR and cC1qR and led to a significant increase in the deposition of C3b compared with tobacco smoke extracts. Also, the activation, aggregation, and adhesion of platelets were markedly enhanced following exposure to ECV. Due to the inhibition of platelet functions after exposure to pure nicotine, it is believed that other components of ECV can antagonize platelet functions. Collectively, e-cigarettes shorten physiological hemostasis and increase the risk of thrombogenic events, which enlightens us to pay attention to the negative impact of e-cigarette exposure on health.

## Conclusions

Since its launch, e-cigarettes have both benefits and risks. Cardiovascular safety is an important consideration in the use of e-cigarettes. The studies involved in this review have shown that e-cigarettes could lead to adverse effects on cardiovascular system through various mechanisms, such as oxidative stress, inflammation, endothelial dysfunction, atherosclerosis, hemodynamic effects, and platelet activity (Fig. 2). Furthermore, flavorings are also one of the important hazards. It is reported that cinnamaldehyde has strong harmfulness. However, considering that there are many flavorings of e-cigarettes on the market and their update is fast, it is not easy to systematically and comprehensively summarize the toxicity of flavorings. Propylene glycol and glycerol produce toxic emissions during heating, and the cardiovascular toxicity of these emissions varies greatly due to the heating process. Propylene glycol and glycerol can produce toxic emissions during heating, and the cardiovascular toxicity of these emissions varies greatly due to the heating procedure. In addition, if the coils of e-cigarettes are heated, it will promote toxic metal exposure. e-cigarettes, as nicotine delivery systems, theoretically have lower concentrations than tobacco cigarettes, so nicotine toxicity is also lower than cigarettes. Based on reviews of toxicity and mechanisms of e-cigarettes, we concluded that e-cigarettes should be safer for CVDs than tobacco cigarettes, but there is still a lack of toxicity comparison in many aspects; especially the molecular and cellular effects caused by ECV are still unclear. It seems like using e-cigarettes to quit smoking is a practical choice. As a result, great caution and hesitation should remain concerning e-cigarette use until its health risk profile is better established. Nowadays, as a tool for smoking cessation, e-cigarettes have led to more and more “dual smokers,” who have a higher risk than using either product alone. Except for smokers, this may pose a serious threat to more vulnerable members, such as children and adolescents, who may be persuaded to start using e-cigarettes, become addicted to nicotine, and eventually start smoking under unproven safety assumptions. Therefore, on the basis of preclinical experiments, more high-quality clinical trials are needed to finally determine the safety and effectiveness of e-cigarettes [21]. In addition, it is important for the health care community to remain informed about the mechanisms of deleterious health outcomes led by e-cigarettes, coupled with behavioral studies, which can better formulate regulatory guidelines defining their proper use and public perception, which is crucial to generate the scientific evidence needed to hone regulatory priorities and justify policy changes to better protect public health.

## Data Availability

No datasets were generated or analysed during the current study.

## Abbreviations

ACS: Acute coronary syndromes
ApoE^−/−^: Apolipoprotein-E knockout
AS: Atherosclerosis
CVD: Cardiovascular disease
DNA: Deoxyribonucleic acid
e-cigarette: Electronic cigarette
ECV: e-cigarette vapor
eNOS: Endothelial nitric oxide synthase
EPA: Environmental Protection Agency
EPCs: Elevated endothelial progenitor cells
HDL: High-density lipoprotein
HUVECs: Human umbilical vein endothelial cells
IHD: Ischemic heart disease
JAK/STAT: Janus kinase/signal transducer and activator of transcription
LDL: Low-density lipoprotein
MI: Myocardial infarction
NHIS: National Health Interview Survey
Nrf2: Nuclear Factor erythroid 2-Related Factor 2
OR: Odds ratio
PI3K: Phosphatidylinositol-3-kinase
p38 MAPK: p38 MAP kinase
ROS: Reactive oxygen species
SCD: Sudden cardiac death
TLR9: Toll-like receptor 9
WHO: World Health Organization
